# The Bombay Medical Congress

**Published:** 1909-03-27

**Authors:** 


### The Hospital

A Journal of

The Medical Sciences and Hospital Administration.

New Series. No. 108, Vol. IV. [No. 1180, Vol. XLV.] SATURDAY, MARCH 27, 1909.

### THE BOMBAY MEDICAL CONGRESS.

The Medical Congress held at Bombay towards the close of last month seems to have been a decided success, although nothing epoch-making transpired in the course of its deliberations. Major Ronald Ross delivered an address upon malaria prevention, advocating mosquito destruction in large towns, where it is more or less easily accomplished; quinine in country districts, where the destruction of mosquitoes offers far greater difficulties; and a combination of both wherever malaria is rampant. Capt. Cristophers and Dr. C. A. Bentley drew attention to the fact that infection is very slowly eliminated from a population living in poverty and hardship. Hence it is not sufficient to pursue the destruction of mosquitoes alone: since in the presence of a people abundantly, though perhaps latently, infected, a supply of anopheles in itself quite small was capable of perpetuating the infection indefinitely. These chronically infected individuals, in fact, form a permanent reservoir of the disease, and are a standing menace not only to their immediate neighbourhood, but to any districts to which their travels chance to take them. The aggregation of communities which is attending the development of the coalfields and other resources of the country may be expected, according to the authors, to accentuate these predisposing factors, unless care is taken to abate them by minimising, as far as is possible, the privations, hardships, and bad hygienic conditions under which the people live. Professor Kitasato communicated a paper on plague, read by Professor Shiga. It showed that the faculty in Japan has now adopted the Indian explanation of the transmission of plague, namely, by fleas, a proposition it has, until lately, been loth to accept. Capt. Liston, I.M.S., spoke enthusiastically of the value of preventive inoculation, declaring that there could be no question of the efficiency of the method. He holds that at least six times as many lives can be saved by this means as can be saved without it.

The discussion upon snake bites and the means of combating them resulted in a general consensus of opinion that the treatment advocated by Sir Lauder Brunton was not to be relied upon. This method, it will be remembered, is carried out as follows: A tight bandage is applied above the bite, and the punctured wound is converted into an incised one with a lancet, permanganate of potash being then thoroughly rubbed into the wound. It was pointed out that in practice the site of deposition of the poison does not always correspond to the position of the skin-wound owing to the free mobility of the skin, and that in consequence it is impossible to be sure that the antidote is being applied to the appropriate spot. While it was not denied that the treatment had in several cases been employed with good results, it appeared to be considered that the claims made for the method had not been satisfactorily established, especially since animal experiments, performed upon monkeys under the most favourable conditions, had failed. The serum treatment of snake-bite also came under review, and again with rather disappointing results. It seems that up to the present it has been impossible to prepare a serum active against all varieties of snake poison. The serum issued from the Kasauli Institute is prepared from a mixture of cobra and daboia venom, and its efficiency is strictly limited to these two varieties of poisoning, while there are thirty-nine known species of terrestrial poisonous snakes in India, exclusive of a variety of sea-snakes.

As regards beri-beri there was little or no unanimity. Mr. Leonard Braddon claimed that the cause of the disease was a specific poison, found in rice alone, and only when the rice is in a certain state of staleness as a result of exposure for some time subsequent to decortication. He admitted that attempts to isolate the hypothetical poison had failed. Subsequent discussion produced instances of the occurrence of the disease apart altogether from the eating of rice, a case being cited of sixty-one British soldiers who developed the malady at Poona within a period of three months. The cause of beri-beri seems to remain as mysterious as ever.

The Nastin treatment of leprosy attracted much attention. A communication from Professor Deycke was read in which he described at length the method by which he isolated the neutral fat to which he has given the name Nastin. Benzoyl-nastin is, he says, an agent which acts directly upon leprosy bacilli.

He does not claim for it an infallibly remedial action, but asserts that it will often ameliorate or even arrest the disease; and a certain amount of confirmation was forthcoming.

A variety of other matters came before the Congress, but their importance was less far-reaching. The précis given above is enough to emphasise the value of such meetings, for in no other way can the experience of practical men be collated from year to year. In the case of tropical medicine such intermittent collation of experience is of particular moment, for in this field many new treatments and new modifications of old treatments, are still on their trial, and it is of the utmost importance that the world should know, at the earliest possible moment, which are worthy of survival and which have been found wanting. The perpetuation of useless treatments is a great drag on progress, and the timely elimination of them is, in itself, no mean service to the cause of medicine.

